# Clinical Cases in Surgical Wards of Mercy Hospital

**Published:** 1871-07

**Authors:** 


### Clinical Reports.

### CLINICAL CASES IN SURGICAL WARDS OF MERCY HOSPITAL.

Service of PROF. ANDREWS. From Notes by M.

CASE I.—TRAUMATIC TETANUS.—RECOVERY.—D. M., aged 36, occupation laborer, while working in a distillery on March 28th, 1871, had his right arm caught in a belt revolving at the rate of 180 revolutions per minute. The tissues of the arm were mutilated to a great extent, and a portion of the peripheral nerves badly lacerated. He was admitted to Mercy Hospital, March 29th, and was placed at rest with good diet, and tinct. ferri chloridi, gtts. 25, every two hours. On March 30th, indications of erysipelatous inflammation were noticed, with sloughing of a gangrenous nature. The patient was suffering greatly, and anodynes were freely given, the tr. iron being continued. Suppuration and sloughing from the wound continued copious until April 17th, when Prof. Andrews amputated the arm. *April 18th.* Tetanus supervened, involving the intercostal muscles of the right side, also of the neck, back, lower limbs, and face, so that he was unable to open the mouth, the muscles of the jaw feeling stiff and rigid. Sulph. quinia was administered freely, 8 grs. every hour, combined with anodynes. Up to April 21st, there was no improvement, the patient gradually failing in strength. Commenced giving tinct. Calabar bean in 10 gtt. doses every four hours alternately with sulph. quinia, 10 grs. *April 22d.* Increased the dose of the tinct. Calabar bean to 15 gtts. every two hours. On the 23d the dose was increased to 40 gtts.—patient slightly better. *April 24th.* Commenced giving the tinct. Calabar bean in teaspoonful doses every four hours alternately with sulph. quinia, 8 grs., and the treatment was thus *heroically* carried out until all rigidity of the muscles had disappeared. *May 10th.* Discontinued all treatment, patient able to walk about the wards.

Tinct. Calabar bean seems to possess the property of relaxing the muscular tissues and subduing the spasms that occur in tetanus.

Case II.—Local Erysipelas (with remote syphilitic taint). Patient admitted April 10th. Face red, and much swollen; high fever, pulse 120 per minute; great prostration; had been sick for several days previous to admission. He was placed upon tinct. ferri chlorid., 25 gtts. every three hours, alternated with soda sulphite, 8 grs. To reduce the fever and rapidity of the circulation the following mixture was ordered:

| | | |
|---|---|---|
| ℞. | Tinct. verat. viride | ʒj. |
| | Camph. tinct. opii | āā ℥jß. |
| | Nitrous ether | |

A teaspoonful to be given four times a day.

*April 15th.* Patient sitting up. Dropped the sedative fever mixture and substituted instead, on account of the syphilitic taint:

| | | |
|---|---|---|
| ℞. | Iod. potass | ʒiij. |
| | Fl. ext. conium | ℥j. |
| | Hydrg. chlor. | 1 gr. |
| | Aqua et syrupus | āā ℥jß. |

A teaspoonful to be taken after meals and at bedtime.

At the expiration of another week the patient was convalescent. *May 1st.* Discharged cured.

Case III.—Carbolated Cerate Dressing in Varicose Ulcer of Leg.—B. F., aged 28, native of Scotland, has been troubled for 15 years with a chronic ulcer on the leg, and with varicose veins in both lower limbs. The ulcer was discharging freely at the time of his admission, May 13th. Prof. Andrews injected several of the varicose veins with tinct. ferri murias, and dressed the ulcer with an antiseptic ointment, composed of carbolic acid cryst., 10 grs., adepis, ℥j. The patient continued to improve from date of admission, with good diet, liberal exercise in the fresh air, etc. *June 1st.* The discharge had entirely ceased from the ulcer, and the patient was discharged cured.

